# The Peyer’s Patch Mononuclear Phagocyte System at Steady State and during Infection

**DOI:** 10.3389/fimmu.2017.01254

**Published:** 2017-10-02

**Authors:** Clément Da Silva, Camille Wagner, Johnny Bonnardel, Jean-Pierre Gorvel, Hugues Lelouard

**Affiliations:** ^1^Aix-Marseille University, CNRS, INSERM, CIML, Marseille, France; ^2^Laboratory of Myeloid Cell Ontogeny and Functional Specialisation, VIB Inflammation Research Center, Ghent, Belgium

**Keywords:** mucosal immunity, Peyer’s patch, dendritic cells, macrophages, M cells, microbiota, IgA, bacterial and viral infections

## Abstract

The gut represents a potential entry site for a wide range of pathogens including protozoa, bacteria, viruses, or fungi. Consequently, it is protected by one of the largest and most diversified population of immune cells of the body. Its surveillance requires the constant sampling of its encounters by dedicated sentinels composed of follicles and their associated epithelium located in specialized area. In the small intestine, Peyer’s patches (PPs) are the most important of these mucosal immune response inductive sites. Through several mechanisms including transcytosis by specialized epithelial cells called M-cells, access to the gut lumen is facilitated in PPs. Although antigen sampling is critical to the initiation of the mucosal immune response, pathogens have evolved strategies to take advantage of this permissive gateway to enter the host and disseminate. It is, therefore, critical to decipher the mechanisms that underlie both host defense and pathogen subversive strategies in order to develop new mucosal-based therapeutic approaches. Whereas penetration of pathogens through M cells has been well described, their fate once they have reached the subepithelial dome (SED) remains less well understood. Nevertheless, it is clear that the mononuclear phagocyte system (MPS) plays a critical role in handling these pathogens. MPS members, including both dendritic cells and macrophages, are indeed strongly enriched in the SED, interact with M cells, and are necessary for antigen presentation to immune effector cells. This review focuses on recent advances, which have allowed distinguishing the different PP mononuclear phagocyte subsets. It gives an overview of their diversity, specificity, location, and functions. Interaction of PP phagocytes with the microbiota and the follicle-associated epithelium as well as PP infection studies are described in the light of these new criteria of PP phagocyte identification. Finally, known alterations affecting the different phagocyte subsets during PP stimulation or infection are discussed.

## Introduction

In mammals, the gastrointestinal mucosa is the largest surface of interaction with the external environment. This ensures an efficient absorption of nutrients, electrolytes, and water but concomitantly it exposes the body to environmental threats through the ingestion of contaminated food or drinks. Thus, the gut represents a privileged site of entry for various pathogen agents, such as protozoa, bacteria, viruses, toxins, or prion. Different mechanisms of defense exist to protect the body integrity against these threats. The efficient and size-selective shield provided by the mucus layer and the glycocalyx above the villous epithelium favors the uptake of small diffusible molecules while preventing microorganisms from reaching the epithelium. Protection is also ensured through secretion of antimicrobial compounds and innate polyreactive and antigen-specific secretory immunoglobulin A (sIgA) in the intestinal lumen. Finally, the intestinal epithelium forms a physical barrier between the lumen and the *lamina propria*. However, pathogens, such as *Salmonella* and *Shigella*, can survive challenging environmental conditions, disrupt the mucus and the continuity of the epithelial barrier, and penetrate the epithelium to reach interstitial tissues (1). It is, therefore, important for the mucosal immune system to be aware of the presence of pathogens as soon as possible. A simple way to achieve this objective is to provide a facilitated access to the gut luminal content toward the mucosal surface at restricted areas distributed regularly along the gastrointestinal tract. The mammal small intestine possesses such specific sentinel sites marked by the presence of lymphoid follicles. Peyer’s patches (PPs) are the most important of these monitoring sites since they are constituted of several clustered B-cell follicles forming domes interspersed with T-cell zones termed interfollicular regions (IFR). While villi are specialized for absorption of nutrients, PPs are dedicated to the sampling of foreign material and to the induction of mucosal immune responses (2–4). Due to the low number of mucus-secreting goblet cells and lack of polymeric immunoglobulin receptor expression in the follicle-associated epithelium (FAE), PP have a reduced mucus layer and no IgA secretion, respectively, which may favor interaction with pathogens (5, 6). Moreover, the FAE is characterized by the presence of specialized epithelial cells termed M cells, which lack a typical brush border and possess a thin glycocalyx that give a better accessibility to large particulate antigens (7–12). The underlying stromal cell network ensures at least in part this specialization of the FAE. Thus, subepithelial stromal cells express high amounts of the cytokine RANKL, which is necessary to both the production of the chemokine CCL20 by the FAE and the development of M cells (13, 14). The latter display specific carbohydrates and receptors that are used as binding sites by pathogens (15–22). Following their adherence to M cells, particulate antigens are rapidly transported from the lumen to the subepithelial dome (SED) or to an invagination of the basolateral membrane of M cells forming a pocket in which phagocytes, T and B cells reside. Importantly, the presence of M cells is critical for the sampling of both commensals and pathogens (17, 23–25). Once delivered into the basolateral pocket or in the SED, uptake, degradation, and presentation of antigens by the mononuclear phagocyte system (MPS), i.e., macrophages (MF) and dendritic cells (DCs), are key steps to induce a mucosal immune response. During infection, subepithelial phagocytes are, therefore, involved both in PP innate defense and in the initiation of the mucosal immune response (11). However, the role of each phagocyte subpopulation in infection has remained elusive due to an absence of consensual phenotype markers for each subset. Studies have indeed pointed out the substantial overlap in several key surface markers between MF and DC (e.g., CD11c, CD11b, SIRPα, and the major histocompatibility complex class II, MHCII) (26). Thus, until very recently, the characterization of MF in PP has been hampered by the lack of reliable markers. Finally, each dome of a given PP is surrounded by villi, thus preventing an easy discrimination of phagocytes from dome and dome-associated villus (DAV). Although IFRs are located on the sides of each dome, we, hereafter, refer FAE, SED, follicle, and IFR-located phagocytes jointly as dome phagocytes by opposition with DAV phagocytes.

In this review, we focus on recent advances, which have allowed distinguishing the different dome mononuclear phagocyte subsets. We provide an overview of their phenotype, distribution, ontogeny, lifespan, transcriptional profile, and function. We then consider some PP functional studies in the context of these new criteria to propose an identification of implicated dome phagocytes. Finally, we discuss alterations affecting the different phagocyte subsets upon PP stimulation or infection.

## Diversity and Specificity of the PP MPS

Recent progresses in the characterization of PP MPS have demonstrated that dome DC and MF display unique characteristics very distinct from their DAV counterparts (Table 1).

**Table 1:** Peyer’s patch (PP) phagocyte subsets at steady state.

PP subset	Phenotype^a^	Minimal markers^b^ for LSM of WT mouse^c^^,^^d^	Renewal rate	Reference
DN cDC2	CD11c^hi^MHCII^+^**SIRPα^+^CD11b**^−^BST2^−^MerTK^−^CD8α^−^CX_3_CR1^−^CD101^−^	SED: **CD11c, SIRPα**, MerTK/lysozyme	Fast	(38, 40)
CD11b^+^ cDC2	CD11c^hi^MHCII^hi^**SIRPα^+^CD11b^int^**BST2^−^MerTK^−^CD8α^−^CX_3_CR1^−^CD101^−^	IFR: **CD11c, SIRPα**, MerTK,/lysozyme	Fast	(38, 40)
CD8α^+^cDC1	CD11c^hi^MHCII^hi^CD8α^+^**XCR1^+^SIRPα**^−^BST2^−^MerTK^−^CD11b^−^ CX_3_CR1^−^CD101^−^	IFR: **CD11c**, SIRPα	Fast	(38, 40)
Plasmacytoid DC	CD11c^int^MHCII^+^SIRPα^int^**BST2^hi^**B220^+^MerTK^−^CD11b^−^CX_3_CR1^−^CD101^−^	IFR: **BST2**	Unknown	(44, 50), this review
LysoDC	CD11c^hi^MHCII^hi^SIRPα^hi^CD11b^hi^**BST2^int^MerTK^+^lysozyme^+^CX_3_CR1^+^CD4**^−^TIM4^−^F4/80^−^	SED, F: **CD11c, MerTK/lysozyme**, CD4	Fast	(39, 42)
TIM^−^4^−^ LysoMac	CD11c^hi^MHCII^lo^SIRPα^hi^CD11b^int to hi^**BST2^int^MerTK^+^lysozyme^+^CX_3_CR1^+^CD4^+^TIM4**^−^F4/80^−^	SED, F: **CD11c, MerTK/lysozyme, CD4**, TIM^−^4	Slow	(39)
TIM^−^4^+^ LysoMac	CD11c^hi^MHCII^lo^SIRPαhiCD11b^int^**BST2^int^MerTK^+^lysozyme^+^CX_3_CR1^+^CD4^+^TIM4^+^**F4/80^−^	IFR, F: **CD11c, MerTK/lysozyme, TIM**^−^**4**	Slow	(39)
TBM	**CD11c**^−^MHCII^−^CD11b^−^SIRPα^+^**MerTK^+^lysozyme^+^CX_3_CR1^+^CD4^+^TIM4^+^**F4/80^−^	GC: **MerTK/lysozyme, TIM**^−^**4**, CD11c	Unknown	(39, 42)
Main DAV conventional DC	CD11c^hi^MHCII^hi^SIRPα^int^CD11b^hi^MerTK^−^**CD101^+^**CX_3_CR1^−^CD8α^−^	DAV: **CD11c, CD101**	Fast	(39, 40)
Main DAV MF	CD11c^hi^MHCII^hi^SIRPα^int^CD11b^hi^**MerTK^+^CX_3_CR1^+^F4/80^+^**lysozyme^−^CD8α^−^CD101^−^	DAV: **CD11c, MerTK/F4/80**	Slow	(39, 40)

*Gray background, common dendritic cell precursor-derived cells; white background, monocyte-derived cells (TBM may also be derived from embryonic precursors)*.

*^a^In bold, main distinctive markers of each subset*.

*^b^The main location is indicated and together with the expression (bold) or not (regular) of each marker it allows the discrimination of the given subset by LSM*.

*^c^The most efficient surface marker panel to discriminate PP phagocyte subsets by flow cytometry up to now is CD11c, MHCII, SIRPα, BST2, CD4, TIM-4, XCR1, CD11b, CD101*.

*^d^In *Cx_3_cr1*-GFP (monocyte-derived cell labeling) or *Zbtb46*-GFP (conventional DC labeling) transgenic mice, MerTK or lysozyme staining can be omitted*.

*LSM, laser scanning microscopy; SED, subepithelial dome; IFR, interfollicular region; GC, germinal center; DAV, dome-associated villus; DN cDC2, double-negative cDC2; TBM, tingible-body macrophages; MF, macrophages; WT, wild type*.

### Dome Conventional DC

**Figure 1:**
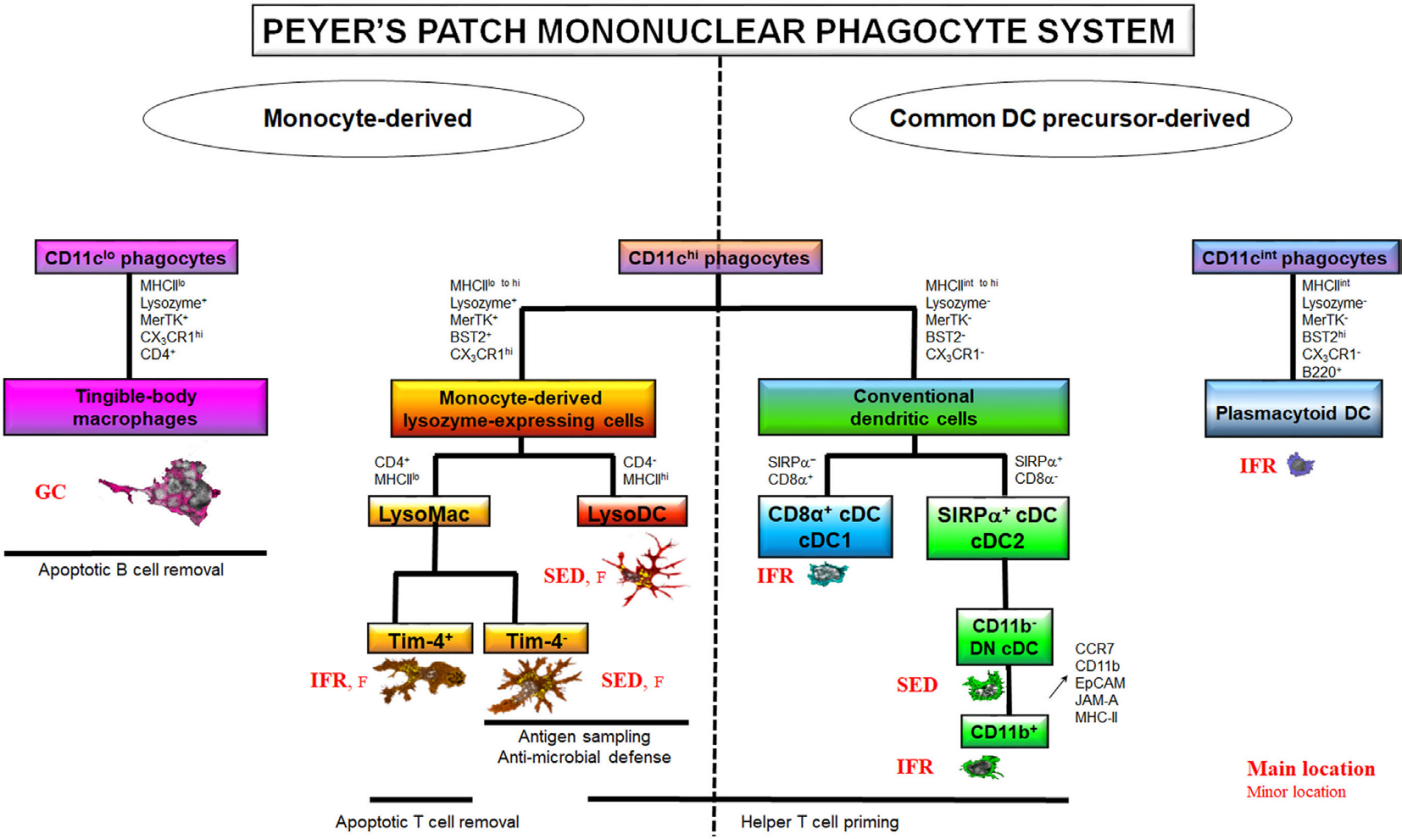
The Peyer’s patch (PP) mononuclear phagocyte system (MPS). The PP MPS encompasses two large families of cells based on their origin, the common DC precursor (CDP)-derived and the monocyte-derived phagocytes. The CDP-derived cells comprise CD11c^hi^ conventional DC (cDC) and CD11c^int^ plasmacytoid DC. Among cDC, cDC1 are CD8α^+^ but SIRPα^−^ whereas cDC2 are SIRPα^+^ but CD8α^−^. cDC2 exist in several stages of differentiation among which the two extremes are the so-called double negative (DN) cDC2, which do not express CD11b, and the CD11b^+^ cDC2. CD11b^+^ cDC2 derive from DN cDC2 through the upregulation of CCR7, CD11b, EpCAM, JAM-A, and MHCII. CDP-derived cells are mainly located in the T cell zones, i.e., interfollicular regions (IFR), at the exception of DN cDC2, which transit through the subepithelial dome (SED). cDC excel in helper T cell priming but are poorly phagocytic. On the contrary, CD11c^hi^ monocyte-derived cells are strongly phagocytic. They also display a broad range of antimicrobial defense mechanisms. CD11c^hi^ monocyte-derived cells encompass two main subsets based on their phenotype, lifespan, and ability to prime T cells: macrophages (MF) and the monocyte-derived dendritic cell (DC) termed LysoDC. LysoDC are CD4^−^MHCII^hi^ short-lived SED-located DC with helper T cell priming ability. CD11c^hi^ MF, also called LysoMac, are CD4^+^MHCII^lo^ long-lived cells without any helper T cell priming ability. TIM-4^−^ LysoMac are mainly located in the SED whereas TIM-4^+^ LysoMac are mainly located in the IFR. A third type of MF, termed tingible-body macrophages, reside in the germinal center (GC) of the follicle (F) where they are involved in apoptotic B cell removal. Unlike other PP MF, they do not express CD11c. Although shown on the monocyte-derived cell part of the diagram, it is currently unknown whether they truly derive from monocytes or whether they self-renew from embryonic precursors. Adapted from Ref. (39).

Mouse common DC precursor (CDP)-derived DC, also called conventional DC (cDC), comprise two major subsets, which have been first identified through the expression of either CD8α (cDC1) or CD11b (cDC2) in addition to CD11c and MHCII (27, 28). Recently, more reliable, specific, and cross-species conserved markers for cDC1, such as XCR1 and Clec9a, have been identified (29–34). Similarly, SIRPα is a more widely distributed marker of cDC2 than CD11b, although shared with MF (35). Both cDC1 and cDC2 are present in domes (Figure 1) (36, 37). In addition, a third cDC subset, termed double negative cDC (DN cDC), which neither expresses CD11b nor CD8α, has been described in PP (37, 38). However, DN cDC have been recently identified as belonging to the cDC2 subset (Figure 1). They indeed share key surface markers with cDC2, such as SIRPα and Clec4a4, and, unlike cDC1, do not depend on Batf3 for their differentiation (39, 40). In addition, the transcriptional programs of CD11b^+^ and DN cDC are very close from each other. Notably, CD11b^+^ cDC express more MHCII at their surface and higher levels of key maturation marker genes such as *Stat4, Ccr7, Ccl22, Socs2*, and *Il6* than DN cDC (40). Moreover, the latter are able to express CD11b upon *in vitro* culture and are recruited in PP before CD11b^+^ cDC (40). Therefore, it is assumed that DN and CD11b^+^ dome cDC represent immature and mature homeostatic differentiation stages of cDC2, respectively. Dome cDC2 encompass actually a developmental continuum of cells with gradual surface acquisition of CCR7, CD11b, EpCAM, JAM-A, and MHCII and decrease of CD24 expression (40). Importantly, dome cDC2 are distinct from DAV cDC2 (Table 1). Thus, the latter display more CD11b and less SIRPα at their surface than dome cDC2. Moreover, most of them express CD101 whereas dome cDC2 do not (40).

### Dome MF

Unlike villous MF, identification of dome MF has remained unsolved for decades due to the lack of expression of classic macrophage markers such as F4/80 (EMR1), sialoadhesin (Siglec1/CD169), Mannose Macrophage Receptor (MMR/CD206), or Fc Gamma Receptor I (FcGRI/CD64) (39). Moreover, a substantial overlap of key surface markers, such as CD11c, CD11b, MHCII, and SIRPα, exists between MF and cDC (26). By the past, this has led to a great confusion concerning location and functions of both dome phagocyte populations. However, recent works managed distinguishing dome cDC from monocyte-derived cells (Table 1) (39–41). The latter encompass dome MF and the monocyte-derived DC termed LysoDC (Figure 1). Most dome monocyte-derived cells express CD11c, CD11b, SIRPα, BST2, CX_3_CR1, MerTK, and lysozyme (Table 1). BST2 and lysozyme expression are hallmarks of dome monocyte-derived cells since DAV MF express little or none of these molecules (39, 42). Thus, CD11c^+^ dome MF have been termed LysoMac by analogy with LysoDC and by opposition to villous MF, which do not express lysozyme. LysoMac are strongly autofluorescent large long-lived cells, which depend on the growth factor M-CSF to develop (39). They express CD4 but only low levels of MHCII. They encompass two main subsets based on the expression of the phosphatidylserine receptor TIM-4 (Figure 1) (39).

Tingible-body macrophages (TBM), which also display TIM-4 at their surface, form a third dome macrophage subset (39). Like LysoMac, TBM express MerTK, CX_3_CR1, SIRPα, lysozyme, and CD4 but typically lack CD11c, CD11b, and MHCII expression (Table 1) (39, 42). BST2 expression in TBM has not been investigated so far. Although easily detectable *in situ* through the presence of many internalized apoptotic bodies, they have been poorly characterized and their origin, either circulating monocytes, embryonic precursors or both, is unknown. In addition to these subsets, PP contain a layer of poorly described serosal/muscularis MF, which, depending on their location, express or not CD169 (see below) (39).

### LysoDC

LysoDC are short-lived monocyte-derived DC (Figure 1; Table 1) (39). Unlike LysoMac, they are weakly autofluorescent, express very high levels of MHCII, but no CD4, and are strongly dependent on CCR2, the chemokine receptor that allows monocyte egress from the bone marrow (39). Morphologically, they are large stellate motile cells (42, 43). Upon stimulation with the TLR7 agonist R848, they secrete IL-6 and TNF but no IL-10 (Table 2) (39). So far, no equivalent of LysoDC has been described in villous *lamina propria*. LysoDC are present in mouse, rat, and human PP (42). Thus, these phagocytes seem to be specific of PP and maybe of isolated lymphoid follicles in several species including humans.

**Table 2:** Functions of dome phagocyte subsets.

Peyer’s patch subset	Antigen sampling activity	Apoptotic cell removal	Cytokine production	T cell priming and polarization	IgA production induction	Reference
Double-negative cDC2 (DN cDC2)	Unknown	Unknown	(*)	(*)	*In vivo* candidate	(38, 39, 89, 90)
CD11b^+^ cDC2	Unknown	Unknown	IL-6	IL-6	*In vivo* candidate	(38, 39, 89, 90)
CD8α^+^ cDC1	Unknown	Unknown	IL-12 p70	IFNγ	No	(38, 39, 89, 90)
Plasmacytoid DC	Unknown	Unknown	IL-12 p70, No type I IFN	IL-17	*In vitro*, not *in vivo*	(44, 45, 87, 88)
LysoDC	Microspheres, *Salmonella*, sIgA-IC	Follicle-associated epithelium (FAE) cells	IL-6, TNF	IFNγ, IL-6, TNF	*In vivo* candidate	(39, 42, 43, 101)
TIM4^−^ LysoMac	Microspheres, *Salmonella*, prion, sIgA-IC	FAE cells	ND	No priming	*In vivo* candidate	(39, 42, 90, 101, 131)
TIM^-^4^+^ LysoMac	Unknown	T cells	ND	No priming	No	(39)
Tingible-body macrophages	Unknown	Germinal center B cells	ND	ND	No	(39, 42)

(*)Upon maturation, DN cDC2 may give rise to CD11b^+^ cDC2 and acquire their functional attributes, i.e., IL-6 production and T cell polarization for IL-6 production.

*Grey background, common dendritic cell precursor-derived cells; white background, monocyte-derived cells*.

*sIgA-IC, secretory immunoglobulin A immune complex; ND, not determined*.

### Plasmacytoid DC

Although PP plasmacytoid DCs (pDCs) share BST2 expression with monocyte-derived cells, they constantly express higher levels of BST2 and lower levels of CD11c and SIRPα than LysoDC and LysoMac (Table 1) (39, 40). One can also distinguish PP pDC from monocyte-derived cells, thanks to their B220 expression. PP pDCs are distinct from pDCs isolated from other tissues by their inability to secrete abundant type I IFN in response to the TLR9 agonist CpG (Table 2) (44). Expression of the mucosal migratory receptor CCR9 and of the specific transcriptional regulator of the pDC lineage E2-2 is also reduced in PP pDCs as compared to other pDCs (45). Like other pDCs, PP pDCs are derived from the CDP and are induced by Flt3L, but their recruitment also requires type I IFN/STAT1 signaling (45). This type I IFN conditioning of PP pDC could favor the production of the inflammatory cytokines IL-6, IL-23, and TNF instead of type I IFNs (45).

## Anatomic Localization of PP Phagocyte Subsets at Steady State

**Figure 2:**
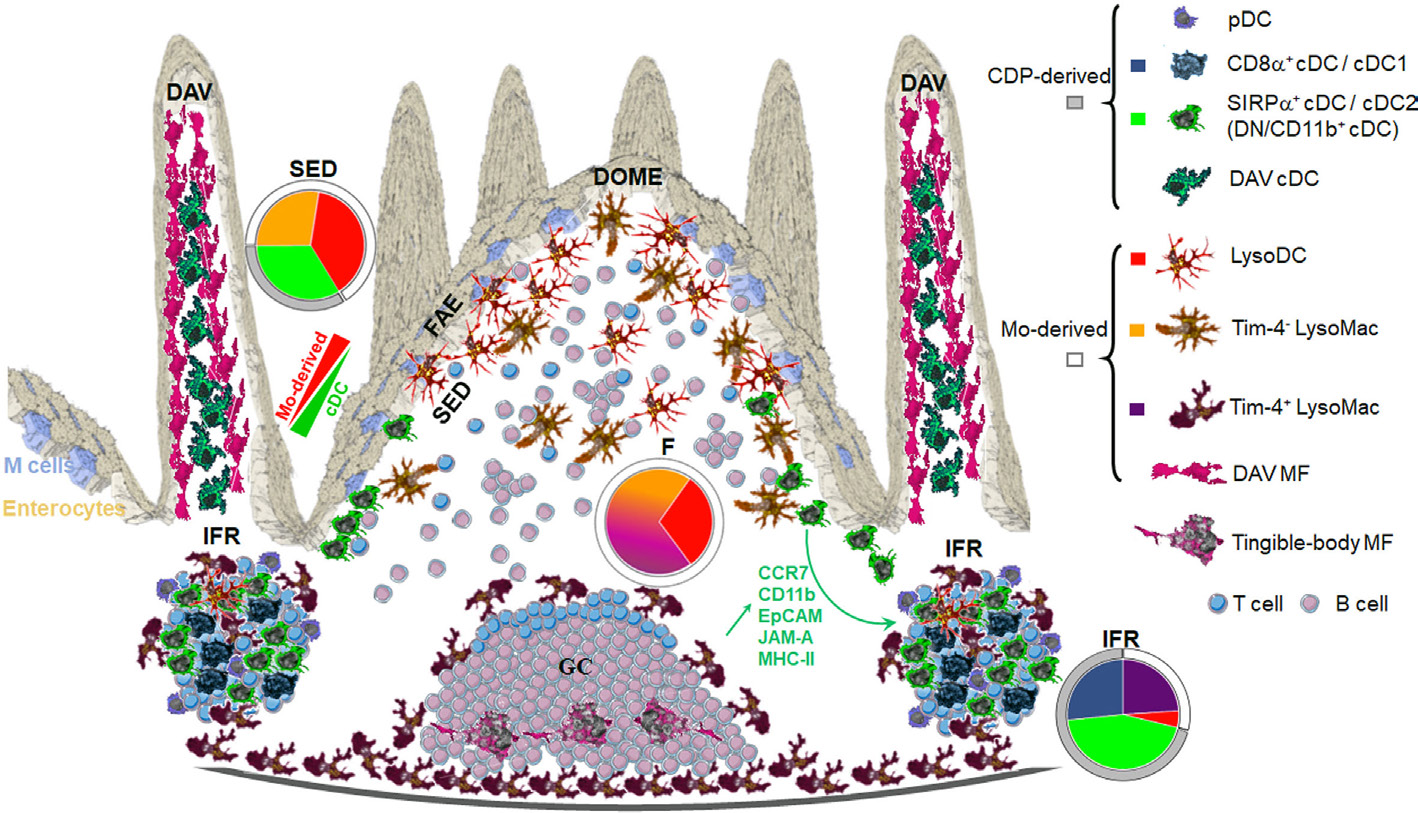
Anatomic localization of Peyer’s patch (PP) phagocyte subpopulations. Origin and shape of each PP phagocyte subset is displayed on the right. Color codes correspond to colors displayed in pie charts. The latter show in each region of the dome the distribution of PP phagocyte subsets at the exception of plasmacytoid DC (pDC). FAE, follicle-associated epithelium; SED, subepithelial dome; F, follicle; IFR, interfollicular region; DAV, dome-associated villus; MF, macrophages. Adapted from Ref. (40).

Each region of the dome, i.e., FAE, SED, follicle, germinal center (GC), and IFR, is populated with specific subsets of phagocytes (Figure 2).

### FAE and SED

**Figure 3:**
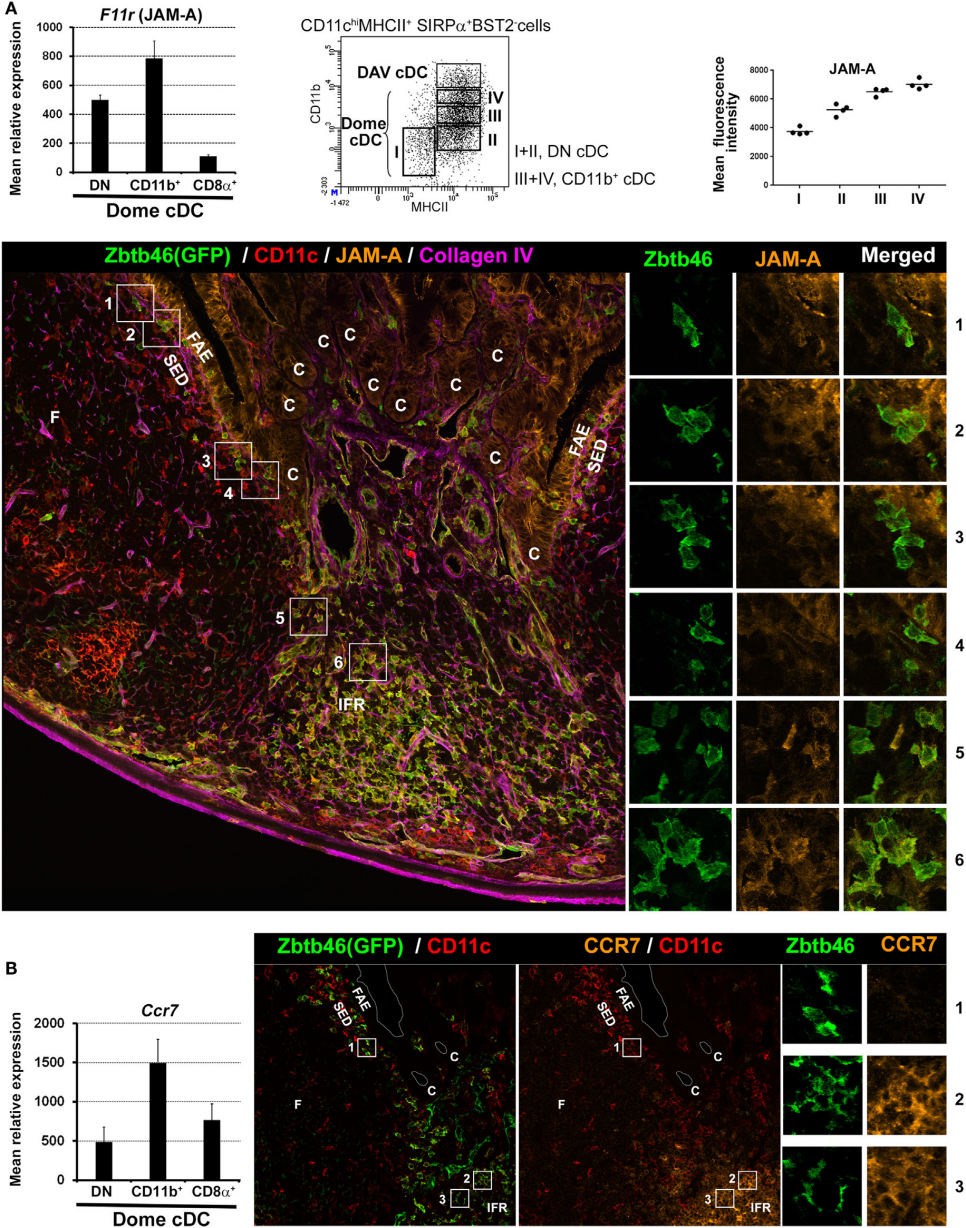
Location of dome double negative (DN) and CD11b^+^ cDC2 based on JAM-A and CCR7 expression. **(A)** Left panel: normalized mean relative expression ± SD of *F11r* (JAM-A) in dome conventional DC (cDC) subsets. Mid-panel: identification of four developmental stages of dome cDC2 based on CD11b and MHCII surface expression. Stage I, CD11b^−^MHCII^lo^; Stage II, CD11b^−^MHCII^int^; Stage III, CD11b^lo^MHCII^hi^; Stage IV, CD11b^int^MHCII^hi^. Right panel: mean fluorescence intensity of JAM-A in the four developmental stages of dome cDC2. JAM-A expression increases from stage I (DN cDC2) to stage IV (CD11b^+^ cDC2). Lower panel: confocal microscopy projection of a *Zbtb46-*GFP^−/+^ mouse Peyer’s patch (PP) section stained for EGFP (green), CD11c (red), JAM-A (orange), and collagen IV (magenta). Higher magnifications of the numbered boxed area are shown on the right. cDC (CD11c^+^GFP^+^ cells) are mainly located in the IFR. However, some of them are located in the SED with a progressive decrease in numbers while reaching the upper part of the dome. Like LysoDC, they can penetrate into the follicle-associated epithelium (FAE). Subepithelial cDC2 (boxed area 1–4) express no or faint levels of JAM-A (stage I or II of dome cDC2; DN cDC2) whereas interfollicular cDC2 (boxed area 5 and 6) express it (stage III or IV of dome cDC2; CD11b^+^ cDC2). **(B)** Left panel: normalized mean relative expression ± SD of *Ccr7* in dome cDC subsets. Right panel: confocal microscopy projection of a *Zbtb46-*GFP^−/+^ mouse PP section stained for EGFP (green), CD11c (red), and CCR7 (orange). Higher magnifications of the numbered boxed area are shown on the right. Subepithelial cDC2 (boxed area 1) do not express CCR7 (DN cDC2) whereas interfollicular cDC2 (boxed area 2 and 3) do (CD11b^+^ cDC2). Parts of **(A,B)** are adapted from Ref. (40).

Subepithelial phagocytes are mainly composed of CD11c^+^ CD11b^+^ cells (37, 40, 42). Due to the expression of both surface markers, these cells were initially thought to be cDC2. However, these subepithelial CD11c^+^CD11b^+^ cells also express CX_3_CR1 and lysozyme and belong to the monocyte-derived family of phagocytes, i.e., LysoDC and LysoMac (40). Both represent actually two-third of subepithelial phagocytes with increasing ratio while reaching the upper part of the dome (Figure 2). By contrast, the SED contains few cDC, mainly DN cDC2 (JAM-A^−^CCR7^−^CD11b^−^SIRPα^+^ cDC), which are rather located in the lower part of the dome (Figure 3) (40). Both subepithelial LysoDC and DN cDC2 can penetrate the FAE and strongly interact with M cells, whereas LysoMac remain mainly located in the SED (40). Heterogeneity in dome macrophage-associated phenotype is tightly linked to their different anatomic localization within PP (Figure 2). This suggests that these phenotype differences reflect an important regional specialization of macrophage functions. Thus, subepithelial LysoMac do not express TIM-4 (39).

### Follicle and GC

Conventional DCs are generally absent from the follicle and from the GC. The upper part of the follicle comprises exclusively scattered LysoDC and TIM-4^−^ LysoMac, whereas in its lower part, TIM-4^+^ LysoMac replace TIM-4^−^ LysoMac (39). Finally, TBM are the only phagocytes of the GC.

### Interfollicular Regions

Interfollicular regions contain mainly cDC1 (SIRPα^−^ cDC), CD11b^+^ cDC2 (JAM-A^+^CCR7^+^CD11b^+^SIRPα^+^ cDC), and scattered TIM-4^+^ LysoMac (Figures 2 and 3) (39, 40). Of note, by microscopy, CD11b staining is not detectable in interfollicular CD11b^+^ cDC2 and TIM-4^+^ LysoMac due to its low levels of expression in these cells (40). Only LysoDC and subepithelial TIM-4^−^ LysoMac actually stain for CD11b in the dome. Since interfollicular cDC (cDC1 and CD11b^+^ cDC2) express CCR7 whereas subepithelial cDC (DN cDC2) do not (Figure 3B), the former are likely recruited through the specific expression of CCL19 and CCL21 in the IFR whereas the latter are likely recruited in the SED through their expression of CCR6 and secretion of CCL20 by the FAE (37, 40, 46–49). Surprisingly, interfollicular TIM-4^+^ LysoMac do not express CCR7, indicating that another factor may be involved in their addressing to the IFR (40). Interestingly, a layer of these MF surrounds the IFR, thus forming border guards of the T cell zone. Finally, CD169 is only expressed by MF located at the base of the IFR toward the serosa, including serosal/muscularis MF (39).

**Figure 4:**
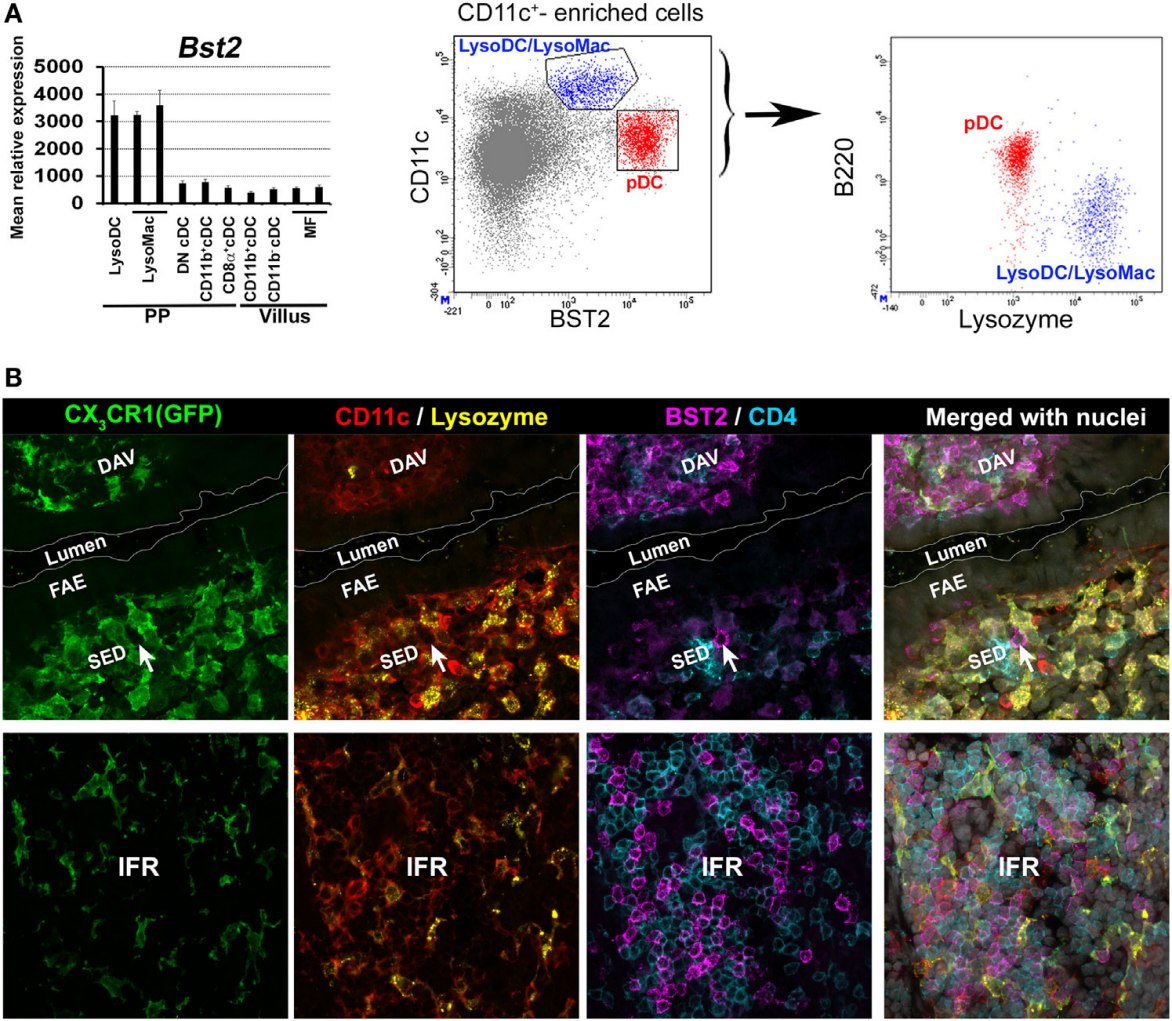
Location of plasmacytoid DC (pDC) in Peyer’s patch (PP). **(A)** Left panel: normalized mean relative expression ± SD of *Bst2* in intestinal phagocytes based on Immgen database (139) and on the PP phagocyte microarray data deposited to NCBI GEO under accession numbers GSE94380 and GSE65514 (39, 40). Expression of *Bst2* by LysoDC and LysoMac is shown. Right panel: in PP, monocyte-derived cells (CD11c^hi^B220^−^lysozyme^+^ in blue), i.e., LysoDC and LysoMac, express lower levels of BST2 than pDC (CD11c^int^B220^+^lysozyme^−^ in red). **(B)** Confocal microscopy projection of CX_3_CR1-EGFP^−/+^ mouse PP sections stained for EGFP (green), CD11c (red), lysozyme (yellow), CD4 (blue), and BST2 (magenta). Upper panel: BST2 is strongly expressed by cells of the dome-associated villus (DAV) but only weakly by LysoDC and LysoMac (CX_3_CR1^+^CD11c^+^lysozyme^+^ cells) in the subepithelial dome (SED). A single putative pDC (arrow) strongly stained for BST2 is located in the SED. Lower panel: unlike the SED, the IFR is enriched in pDC. **(A)** is adapted from Ref. (39).

BST2 has been extensively used to identify pDC in different mouse organs including PP (44, 50). Based on this marker, pDCs were first supposed to be located in the SED and in the IFR (44, 50). However, PP monocyte-derived cells express BST2 at steady state (Figure 4A) (39). Moreover, stimuli that trigger interferon responses induce BST2 expression in several cell types (51). We, therefore, decided to re-investigate pDC location. We found that pDCs are mainly located in the IFR but not in the SED where BST2 is weakly displayed by monocyte-derived cells (Figure 4B).

## Functions of PP Phagocyte Subsets

### Interaction with the FAE and Antigen Sampling Activity

**Figure 5:**
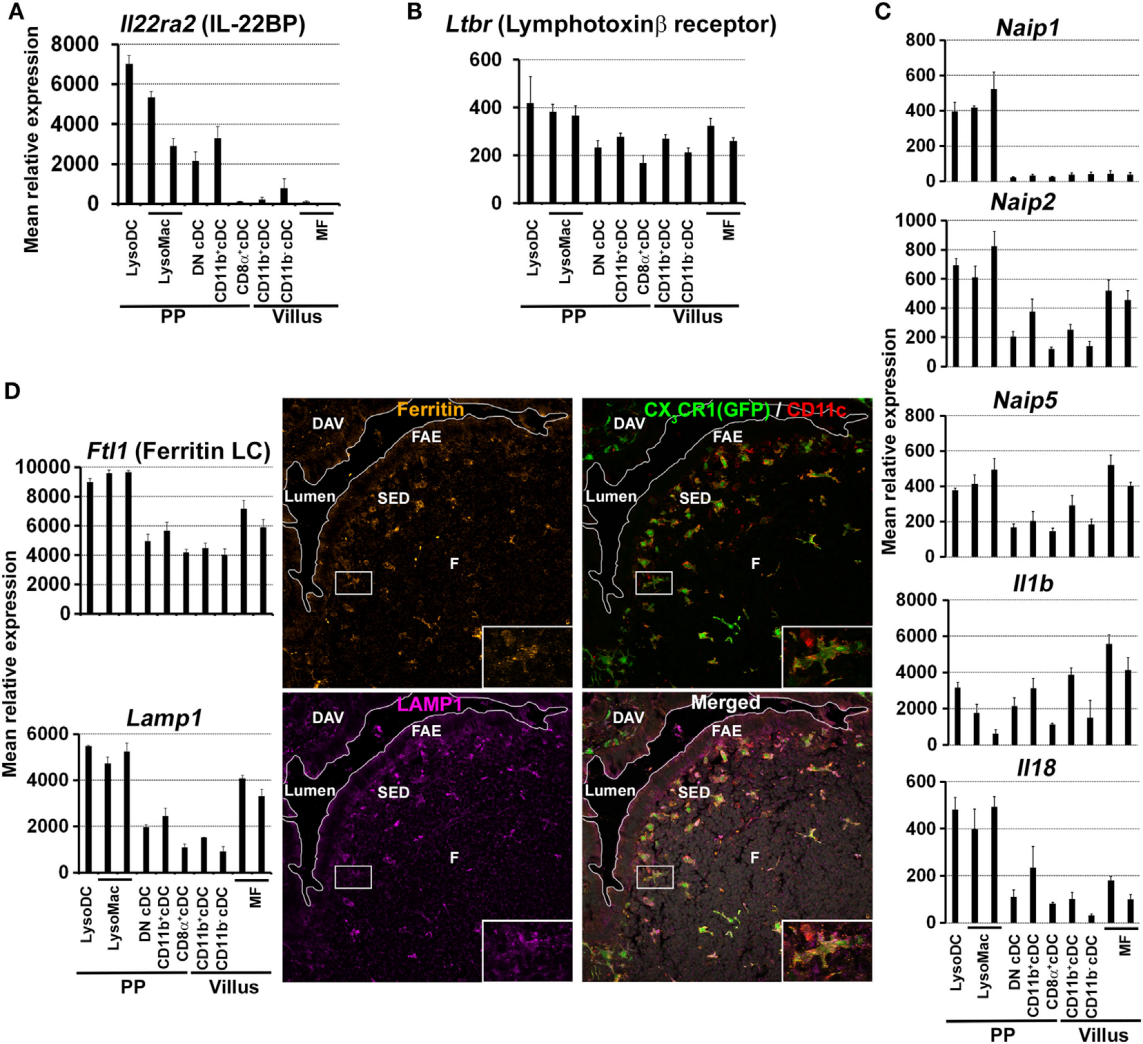
Expression of defined markers by Peyer’s patch (PP) phagocyte subpopulations. **(A–D)** Normalized mean relative expression ± SD of *Il22ra2, Ltbr, Naip1, Naip2, Naip5, Il1b, Il18, Ftl1*, and *Lamp1* in intestinal phagocytes based on Immgen database (139) and on the PP phagocyte microarray data deposited to NCBI GEO under accession numbers GSE94380 and GSE65514 (39, 40). **(A)** In the gut, *Il22ra2* [IL-22 binding protein (IL-22BP)] is mainly expressed by LysoDC and TIM-4^−^ LysoMac. **(B)** In PP, *Ltbr* (lymphotoxin β receptor) is mainly expressed by LysoDC, LysoMac and, to a lesser extent, conventional DC (cDC). **(C)** Enrichment of some members of the NAIP/NLRC4 inflammasome pathway (*Naip1, Naip2, Naip5, Il1b*, and *Il18*) in LysoDC and LysoMac. Note that *Naip1* is only expressed by dome monocyte-derived cells. **(D)** Left panel: in the gut, LysoDC and LysoMac express higher levels of *Ftl1* (ferritin light chain) and *Lamp1* than other phagocytes. Right panel: labeling of a PP section shows enrichment of ferritin and LAMP1 expression in LysoDC and LysoMac of the subepithelial dome (SED) and of the follicle (F). Inserts: higher magnification of the boxed area showing one LysoDC strongly stained for ferritin and LAMP1.

The preferential uptake of luminal particulate antigens in PP as compared to villi first relies on the specific characteristics of the FAE (5–10, 52). Some of these properties, such as low levels of mucin expression, altered surface glycosylation, and lack of secretion of antimicrobial proteins, depend on IL-22 signaling inhibition through the production of IL-22 binding protein (IL-22BP) by CD11c^+^CD11b^+^MHCII^+^ cells of the SED (53). Thereby, IL-22BP promotes microbial uptake into PP by influencing the FAE transcriptional program. Unfortunately, the markers used to identify IL-22BP-secreting cells do not allow distinguishing cDC from monocyte-derived cells (53). In order to better characterize these IL-22BP-secreting phagocytes, we decided to interrogate the gene expression database of dome CD11c^hi^ phagocytes (NCBI GEO accession numbers GSE94380 and GSE65514) for IL-22BP transcripts (*Il22ra2*). *Il22ra2* was enriched in LysoDC and TIM-4^−^ LysoMac as compared to cDC (Figure 5A). These results, together with the preferential location of LysoDC and TIM-4^−^ LysoMac in the SED, support their role in the secretion of IL-22BP, which in turn inhibits IL-22 signaling, alters the FAE transcriptional program, and favors the internalization of both commensal and pathogenic bacteria (53).

**Figure 6:**
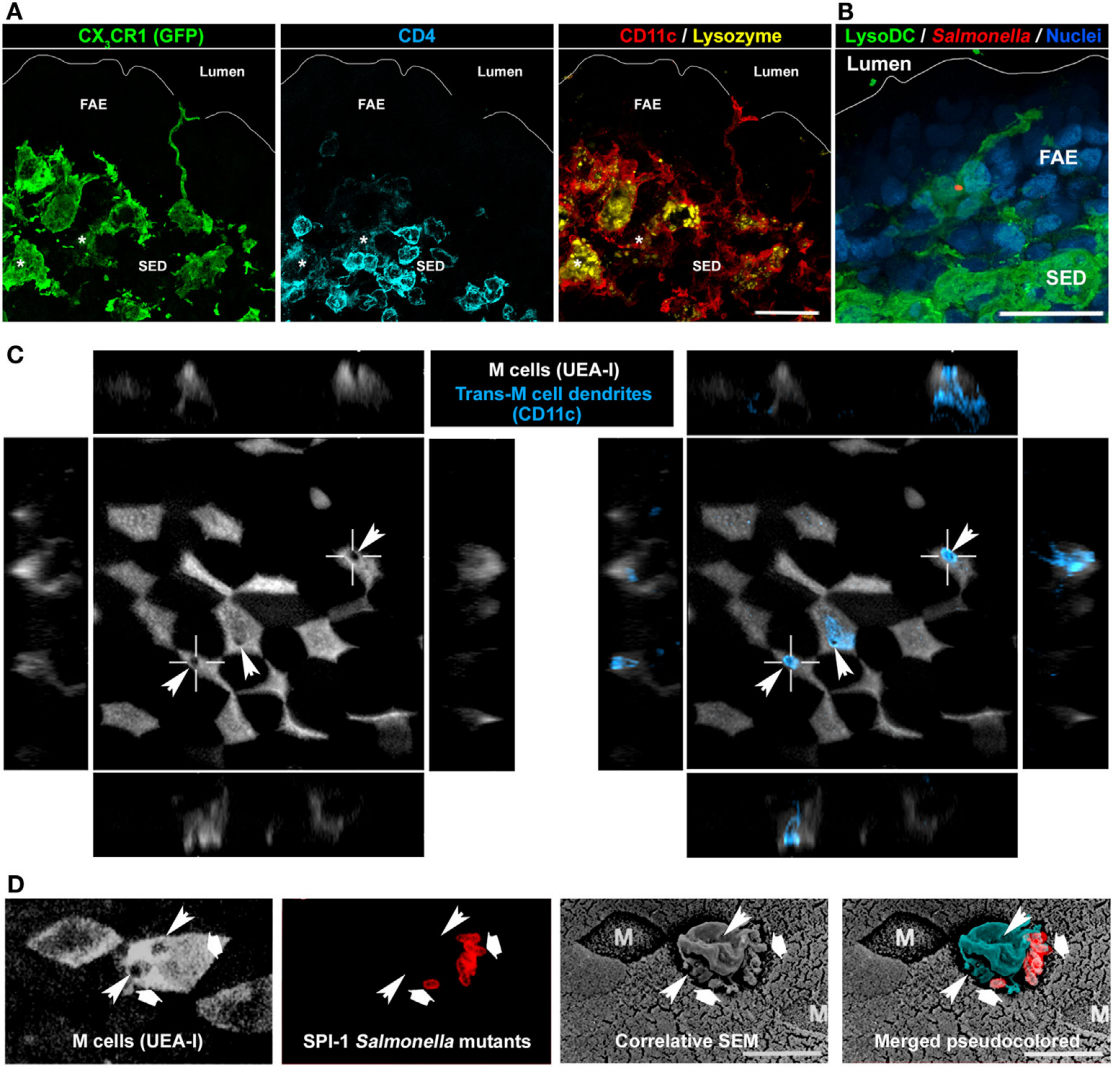
Involvement of LysoDC trans-M cell dendrites in the sampling of *Salmonella typhimurium*. **(A)** A CX_3_CR1-deficient LysoDC (GFP in place of CX_3_CR1 in green; CD11c in red; lysozyme in yellow) extends a dendrite through the follicle-associated epithelium (FAE) to reach the lumen. In addition to CX_3_CR1, CD11c, and lysozyme, LysoMac (*) stain for CD4 (blue). **(B)** 2 h postoral infection, a *Salmonella* (red) is taken up by a LysoDC (green) extending a dendrite into an uninfected FAE. **(C)** M cell transcellular pores (arrowheads), through which trans-M cell dendrites (CD11c in blue) cross the FAE, are highlighted by circular holes in the UEA-I cell surface staining. **(D)** By correlative scanning electron microscopy (SEM), *Salmonella* (second and last panels in red, large arrows) are located at the periphery of a protrusion (pseudocolored in blue) arising from an M cell. Circular holes (thin arrowheads) in the UEA-I cell surface staining indicate the presence of M cell transcellular pores. **(B,C)** are adapted from Ref. (43), **(D)** is adapted from Ref. (110).

In addition to this strong influence on FAE global characteristics, monocyte-derived cells and especially LysoDC maintain privileged interaction with M cells. Thus, LysoDC are able to extend dendrites through M cell specific transcellular pores to gain access to the lumen (Figure 6) (43). The cell adhesion molecules EpCAM and JAM-A are recruited at the M cell pore-forming membrane but neither the tight junction proteins ZO-1 and occludin nor the adherens junction proteins E-cadherin and β-catenin. Therefore, the formation of these M cell transcellular pores does not alter the integrity of the epithelial barrier. JAM-A is also enriched at the trans-M cell dendrite (TMD) membrane, which may favor homotypic interaction. In addition, there is a strong recruitment of filamentous actin in TMD in agreement with their high level of motility. These TMD scan indeed rapidly the surface of M cells and attract particulate antigens and bacteria from the lumen to capture them (43). Since blockade of the M cell-specific chemokine CCL9 drastically reduces the number of CD11c^+^CD11b^+^ cells in the SED and since LysoDC strongly express its receptor CCR1, it is tempting to speculate that the degree of interaction between M cell and LysoDC is regulated through the release control of this chemokine by M cells (39, 49).

Although LysoDC are the main TMD-forming phagocytes, subepithelial LysoDC and TIM-4^−^ LysoMac equally internalize particulate antigens (Table 2) (39). This suggests that, at least at steady state, the main route of particulate antigen sampling across the FAE is mediated through M cell transcytosis rather than by TMD. The lack of particulate antigen uptake by subepithelial cDC (DN cDC2) *in vivo* is in agreement first with their low number in this region and second with *in vitro* microparticle uptake assays showing a much more efficient phagocytic activity of LysoDC and LysoMac as compared to dome cDC (40, 42). Interestingly, in addition to transporting luminal antigens in their basolateral pocket or in the SED, M cells constitutively release on their basal side microvesicles, which are taken up by subepithelial CD11c^+^CD11b^+^CX_3_CR1^+^ cells, i.e., LysoDC and TIM-4^−^ LysoMac (54). Finally, monocyte-derived cells and M cell cooperation extend beyond cell death since the former engulf dying M cells (Table 2) (42). In summary, particulate antigen uptake in the SED is mainly performed by subepithelial monocyte-derived cells and occurs through at least four distinct mechanisms, which all involve M cells: (i) M cell-mediated transcytosis; (ii) M cell microvesicle shedding; (iii) formation of TMD; (iv) dying M cell phagocytosis.

### Innate Defense Functions

LysoDC and LysoMac have been first identified through their strong expression of the antibacterial compound lysozyme (42). Then, they have been distinguished from dome cDC by their surface expression of the host antiviral restriction factor BST2 (39). This suggests that, in addition to playing a primary role in antigen sampling, this family of dome phagocytes are strongly involved in the innate defense of PP. Analysis of their transcriptional profile confirmed this assumption (39). Dome monocyte-derived cells display indeed a strong antibacterial and antiviral gene signature as compared to cDC. This includes genes encoding for viral and bacterial-associated molecular pattern recognition molecules such as toll-like receptors (TLRs), NAIPs, STING, DAI, and RIG-I. Several pathways of antiviral and antibacterial defense such as replication inhibition, metal sequestration, NLRC4 inflammasome formation, and detoxification mechanisms are also upregulated in monocyte-derived cells as compared to cDC. Therefore, LysoDC and LysoMac, but not cDCs, display strong innate defense mechanisms against both viral and bacterial infections (Figure 1).

### Priming of T Cells

Conventional DC have been long recognized as the most efficient professional antigen-presenting cells to initiate an antigen-specific immune response through the priming of both naïve CD4^+^ and CD8^+^ T cells (55). Upon activation, cDC initiate a process of differentiation, also termed maturation, which involves an important genetic reprogramming (56, 57). This induces profound phenotypic, morphological, and functional changes, which allow their migration to lymph node T cell zones and their antigen presentation and naïve T cell priming ability. Depletion of CD11c^+^ phagocytes showed that in PP they are involved both in the retention of interfollicular naive helper T cells and in their priming following antigen feeding (58, 59). Most dome cDC reside in the IFR and, therefore, do not require to migrate to encounter naïve T cells (40). Although it may be convenient and secure to rapidly prime naïve T cells, it raises indubitably the question of antigen acquisition. It could be, however, hypothesized that, after acquisition of soluble antigens or transfer of particulate antigens from monocyte-derived cells, DN cDC2 upregulate CCR7 and downregulate CCR6 to rapidly shuttle from the SED to the IFR. Their constant migratory activity would thus lead to the apparent underrepresentation of cDC in the SED where most of the luminal antigen sampling activity is performed (39, 40). By contrast, LysoDC, which are highly efficient in particulate antigen sampling, are mainly located in the SED and, to some extent, in the follicle but their migration to the T cell zone or their ability to prime T cells *in vivo* in the SED remain pending issues (39, 42, 43).

*In vitro*, unlike LysoMac, both dome cDC and LysoDC are able to induce naïve antigen-specific T helper cell proliferation (Table 2) (39). This is in line with the fact that, upon TLR7 stimulation, LysoDC upregulate MHCII and the co-stimulatory molecules CD40 and CD86 whereas LysoMac do not. Both cDC1 and LysoDC prime naïve antigen-specific T helper cells for IFNγ production. LysoDC also induce the production of IL-6, a property shared with cDC2 (Table 2).

### Interaction with the Microbiota and Induction of the Mucosal Humoral Immune Response

Peyer’s patches are the primary site of antigen-specific sIgA-secreting cell induction (3, 4, 60–64). Production of sIgA is rapidly induced upon microbial colonization and strongly reduced in germ-free animals (65). Moreover, sIgA coating of the microbiota plays a critical role in its diversification (66–68). Interestingly, sIgA predominantly target specific members of the microbiota, especially those residing in the small intestine and those considered as pathobionts (69–71). Therefore, microbiota influences the mucosal immune system, which in turn regulates symbiont diversity and stability (72, 73). Among commensal bacteria that profoundly influence the mucosal immune system in mouse, segmented filamentous bacteria (SFB) play a privileged role, through induction of Th17 cells and IgA-secreting cells (74–76). This SFB-induced immune response largely depends on the stimulation of PPs and isolated lymphoid follicles (77). Interestingly, specific members of the microbiota, including SFB, colonize PPs (77–80). Moreover, M cells are able to transport different defined commensal bacteria, which induce distinct M cell transcriptional programs (81). This sampling of gut microbiota through M cell-mediated pathways is crucial to initiate mucosal sIgA production (25). Thus, it is tempting to speculate that microbiota members capable of inducing strong humoral immune responses are those that strongly interact with the FAE. Once translocated in the SED, SFBs are internalized by CD11c^int^CD11b^+^ and CD11c^hi^CD11b^+^ phagocytes (82). Other IgA-inducer commensals, especially *Alcaligenes* species, reside inside CD11c^+^ cells within isolated lymphoid follicles, PP, and mesenteric lymph nodes (MLN) of mice and humans (79, 80, 83). PP CD11c^+^ phagocytes are known to carry commensal bacteria through a CCR7-dependent mechanism to the MLN, which are required to restrict commensal-loaded CD11c^+^ phagocytes to the mucosal compartment but are dispensable for IgA induction (84). However, the accurate identification of the CD11c^+^ phagocyte subset(s), which sample(s) and carry(ies) commensal bacteria, remains currently unknown.

*In vitro*, the role of PP phagocytes in IgA class switching has long been recognized (85, 86). More recently, it has been shown that PP and MLN pDC efficiently promote IgA class switch recombination through their expression of membrane-bound BAFF (B-cell activating factor) and APRIL (a proliferation-inducing ligand) independently of any T-cell or microbial stimulus (Table 2) (87). However, a recent pDC depletion study showed that pDCs are dispensable for intestinal IgA production *in vivo* (88). CD11c^hi^CD11b^+^B220^−^ phagocytes have also been implicated in the differentiation of naïve B cells into sIgA-secreting cells *in vitro* (89). It remains, however, to establish whether these CD11c^hi^CD11b^+^B220^−^ phagocytes are LysoDC, LysoMac, or dome CD11b^+^ cDC2 and to confirm their function *in vivo*. The role of each dome phagocyte subset in sIgA-secreting cell commitment *in vivo* is indeed not well-established (Table 2). However, efficient IgA class switching requires interaction of CCR6^+^ B cells with lymphotoxin-dependent CD11c^hi^MHCII^+^CD11b^+^ phagocytes in the SED (90). Since lymphotoxin β receptor transcripts (*Ltbr*) are expressed by monocyte-derived cells and, to a lesser extent, by cDC (Figure 5B), it remains to establish whether these CD11c^hi^MHCII^+^CD11b^+^ phagocytes correspond to CD11b^+^ cDC2, LysoDC, or LysoMac. Nevertheless, the sampling activity and the anatomic localization of the different phagocyte subsets as described above would argue for LysoDC/LysoMac rather than for CD11b^+^ cDC2 involvement. The lymphotoxin required to maintain these phagocytes in the SED could be mainly provided by subepithelial innate lymphoid cells type 3 (ILC3) (90). However, a recent report indicates that microbiota-derived butyrate suppresses ILC3 in terminal ileal PP, rendering their role in subepithelial phagocyte maintenance unlikely at least in this part of the gut (91). Lack of CCR6 expression by B cells or RANKL expression deficiency in subepithelial stromal cells, which results in inhibition of CCL20 production by the FAE, prevents B cells migration into the SED, precludes their interaction with CD11c^hi^MHCII^+^CD11b^+^ phagocytes and finally inhibits IgA class switching and bacteria-specific sIgA production (14, 90). The integrin complex αvβ8 expressed by CD11c^hi^MHCII^+^CD11b^+^ phagocytes could directly activate TGFβ during the interaction of these phagocytes with CCR6^+^ B cells and promote IgA class switching (90). In summary, induction of commensal bacteria-specific sIgA-secreting cells is a complex process involving many different cell types: (i) subepithelial stromal cells producing RANKL for the formation of M cells and for the production of CCL20, which recruits CCR6^+^ B cell and DN cDC2 into the SED; (ii) M cells for antigen sampling; (iii) lymphotoxin-producing cells for the maintenance of CD11c^hi^MHCII^+^CD11b^+^ phagocytes; (iv) CD11c^hi^MHCII^+^CD11b^+^ phagocytes for activation of CCR6^+^ B cells.

### Regulation of the Adaptive Immune Response

Tingible-body macrophages are critical in the removal of apoptotic B cells during the selection process that occurs in the GC (Table 2) (92). Defect in this scavenging function leads to secondary necrosis, release of noxious molecules and pro-inflammatory signals, and is linked to autoantibody production and autoimmune disease development. This scavenging function requires the expression of the apoptotic receptor MerTK by TBM and of the soluble bridging molecule MFG-E8 by follicular DC, the GC stromal cells involved in the shaping of the B cell response (93–96). A number of other factors and receptors, such as TIM-4, have also been implicated in this process and their deficiency leads to autoimmunity, too (92). Therefore, although some of these molecules may have redundant roles, they may also function together to be more efficient in apoptotic cell removal through several mechanisms, thus preventing the arising of autoimmunity (97).

Like TBM, interfollicular MF express the apoptotic cell receptor TIM-4 and are located in a region of effector cell priming (39). Although the function of these TIM-4^+^ LysoMac remains elusive, they are thus likely to participate to the clearance of dying T cells like TBM contribute to the removal of dying B cells. Interestingly, TIM-4 deficiency leads to not only B cell but also T cells hyperactivity (98). Moreover, TIM-4 functions have been linked to the control of the adaptive immune response and tolerance through the removal of antigen-specific T cells (99, 100). Concerning PP phagocytes, while LysoDC interact with and prime naïve helper T cells *in vitro*, in the same conditions LysoMac phagocytize them, supporting their role in the removal of T cells (Table 2) (39). Thus, the location of TIM-4^+^ LysoMac in the T cell zone correlates well with a possible function in the removal of some naïve T cells during their priming process. Therefore, TBM and TIM-4^+^ LysoMac could perform a crucial role in PP adaptive immune response regulation at the level of GC and IFR, respectively.

## PP Phagocytes in Infection

### Sensing and Uptake of Immune Complexes

Innate polyreactive and antigen-specific sIgA are secreted by *lamina propria* plasma cells and transported through the epithelium by the polymeric immunoglobulin receptor to be finally released in the lumen. During infection, sIgA recognize and bind pathogens, thus participating to their clearance through a process called immune exclusion. Interestingly, M cells express on their surface dectin-1 and Siglec-F, which can serve as sIgA receptors allowing the uptake of luminal sIgA immune complexes (101). Since uptake of sIgA-coated bacteria persists in PP of dectin-1-deficient mice, Siglec-F expression may be sufficient to mediate sIgA binding to M cells (102). In the SED, sIgA immune complexes are associated with CD11c^+^CD11b^+^CX_3_CR1^+^MHCII^+^ cells, i.e., LysoDC and/or TIM-4^−^ LysoMac (101). Entry of IgA-coated bacteria into PP does not require CX_3_CR1 expression (102). However, this does not preclude a potential role of TMD in sIgA immune complex uptake since CX_3_CR1 is not involved in TMD formation (Figure 6A) (39). Finally, this mechanism of immune complex sampling ensures the constant monitoring of sIgA-coated antigens present in the gut, including both pathogens and symbionts (103, 104). Through this process, new antigen-specific IgA-secreting cells could be produced to allow a better exclusion of pathogens.

### Bacterial Infection: The Case of *Salmonella*

*Salmonella enterica* is an enteroinvasive bacterium typically acquired by ingestion of contaminated water or food. In the absence of dysbiosis, the primary invasion sites of *Salmonella enterica* serovar Typhimurium, the murine model of systemic salmonellosis, are PP of the distal ileum and cecal patches (105, 106). Through their fimbrial FimH adhesin, *Salmonella Typhimurium* are able to bind to the GP2 molecules expressed at the surface of M cells (17). Then, they use their *Salmonella* pathogenicity island 1 (SPI-1) type III secretion system to deliver effector proteins, which reorganize the cytoskeleton and allow the translocation of bacteria through M cells by inducing membrane ruffling (17, 107, 108). However, even SPI-1 and FimH *Salmonella* mutants or GP2-deficient mice show, to some extent, bacterial translocation into PP (17, 102, 109–111). One possible mechanism for this residual penetration could be transcytosis mediated by M cells, as observed with inert particles (13, 112, 113). Alternatively, these bacteria could be directly sampled by TMD (Figure 6) (43). *Salmonella Typhimurium* indeed induce TMD that rapidly internalize bacteria before retracting back to the SED (43). As soon as 2 h after oral infection, bacteria are present in LysoDC extending dendrites into the FAE in absence of any bacterial invasion of epithelial cells (Figure 6B). Interestingly, similar trans-M cell passages of uncharacterized leukocytes have been observed by electron microscopy using rabbit intestinal loop models of *Streptococcus pneumoniae* and *Vibrio cholerae* infection (114, 115). TMD are, therefore, infection-inducible and transient processes, which allow a fast M cell-regulated uptake of luminal material by immunocompetent cells. Such mechanism of sampling may notably avoid risks of massive penetration by pathogens. A typical feature of TMD formation is the appearance of a circular hole at the M cell apical membrane (Figure 6C). Interestingly, correlative scanning electron microscopy studies of PP in mouse intestinal loop infected with SPI-1 *Salmonella* mutants, which are unable to induce epithelial cell apical membrane ruffling, have highlighted the formation of membrane protrusions above M cell surface circular holes (Figure 6D) (109, 110). Therefore, these protrusions, which do not express the M cell marker UEA-I but bind bacteria, are likely TMD.

Once translocated by M cells or internalized by TMD, *Salmonella Typhimurium* are predominantly found in subepithelial lysozyme-expressing cells, i.e., TIM-4^−^ LysoMac and/or LysoDC (Table 2) (42). Importantly, these phagocytes express genes involved in innate defense against *Salmonella* (39). These genes notably include *Naip1, Naip2*, and *Naip5*, which encode cytosolic receptors for the needle and inner rod proteins of the type III secretion system and for flagellin, respectively (Figure 5C) (116). Upon recognition of their ligands, NAIP proteins co-oligomerize with the adaptor NLRC4 to form an inflammasome complex and to recruit and activate caspase-1, which in turn process IL-1β and IL-18 into their active form. Interestingly, monocyte-derived cells express high levels of *Il1b* and *Il18*, indicating that, upon inflammasome activation, they may secrete large amounts of these pro-inflammatory cytokines (Figure 5C). Despite the expression of all these defense genes, it is currently unknown whether TIM-4^−^ LysoMac and/or LysoDC are able to kill internalized *Salmonella* and die from pyroptosis upon inflammasome activation or whether bacteria have evolved strategies to survive, replicate inside, and kill these phagocytes.

Invasion of PP by *Salmonella* also induces the CCR6-dependent recruitment of CD11c^+^ cells in the SED and the FAE, probably through the release of CCL20 by the latter (117). As mentioned above, CCL20 is indeed specifically expressed by the FAE, thanks to its contact with RANKL-producing stromal cells (14). Among PP CD11c^+^ phagocytes, CCR6 expression is restricted to cDC2, and more specifically, DN cDC2 (40, 118). Thus, *Salmonella* induce the recruitment of DN cDC2 in the SED and the FAE. CCR6 expression also promotes the activation of *Salmonella*-specific T cells upon infection (117). Whether this activation relies on the cooperation between monocyte-derived cells that internalize bacteria and DN cDC2, which are recruited to the SED, remains to establish. Interestingly, upon inflammasome activation, pyroptosis of monocyte-derived cells could lead to the release of bacterial antigens and presentation of the latter to T cells by cDC2 as demonstrated in *in vitro* models using mouse bone marrow-derived DC and MF (119). However, since CCR6 is expressed by many other PP immune cell types and is involved in many cellular processes such as B cell migration in the SED and M cell differentiation, it remains to establish whether CCR6^+^ DN cDC2 are directly involved in activation of *Salmonella-*specific T cells upon infection (90, 118, 120–122).

### Viral Infection: Reovirus and Norovirus

Reovirus enters the host through intestinal M cells and lack of M cells prevents from productive infection (18, 23, 123). Similarly, norovirus infection is reduced in M cell-deficient mice (23). Thus, M cells represent preferential entry sites for viruses, in addition to enteropathogenic bacteria. Although noroviruses have a tropism for DC and MF, their precise target following transport through M cells is currently unknown (124, 125). Unlike norovirus, reovirus preferentially replicates within the FAE (126). Interestingly, CD11c^+^ cells of the SED internalize reovirus-infected apoptotic FAE cells (127). As mentioned above, lysozyme-expressing CD11c^+^ cells, i.e., LysoDC and TIM-4^−^ LysoMac, internalize apoptotic FAE cells (Table 2), suggesting their involvement in apoptotic epithelial cell-derived viral antigen handling (42). Importantly, PP CD11c^+^ cells from infected mice are able to process and present viral antigens from apoptotic cells to activate reovirus-primed T cells (127). Finally, initiation of an anti-reovirus immune response characterized by the production of virus-specific sIgA and cytotoxic T cells occurs in PP.

### Prion Infection

Infectious prions are proteins with an abnormal conformation, which, upon conversion of the normally folded endogenous cellular prion protein and spreading to the central nervous system, lead to neurodegenerative diseases. Natural infection occurs mainly by oral consumption of prion-contaminated food. After oral exposure, uptake of infectious prions by M cells and their accumulation and replication upon follicular DCs in small intestine PP are essential for the efficient spread of disease to the brain (128, 129). CD11c^+^ cells are also required for the early stage of PP infection (130). In the SED, infectious prions are located in cells enriched for ferritin and LAMP1 but not MHCII (131). To better characterize these subepithelial phagocytes, we examined the expression of ferritin and LAMP1 transcripts in the gene expression database of dome phagocytes. We found that these transcripts are enriched in LysoDC and LysoMac as compared to cDC (Figure 5D). We also confirmed by immunostaining of PP sections the increased expression of ferritin and LAMP1 inside subepithelial LysoDC and LysoMac (CD11c^+^CX_3_CR1^+^ cells) as compared to other cells (Figure 5D). Since LysoDC express high levels of MHCII, this rather supports a role of TIM-4^−^ LysoMac in the transmission of infectious prion (Table 2). Another population of infectious prion-loaded CD11b^+^ phagocytes is located in the subfollicular area of PP (132). These subfollicular phagocytes are absent from uninfected animals, which suggests that TIM-4^−^ LysoMac could migrate from the SED to this subfollicular area upon infection. Interestingly, CXCR5 expression deletion in CD11c^+^ cells delays accumulation of infectious prion upon follicular DC and impedes oral prion disease pathogenesis (133). This suggests that CXCR5 could allow migration of CD11c^+^ phagocytes from the SED to the follicle or subfollicular area, which would promote spreading of infectious prion to follicular DC. However, when we interrogated the gene expression database of dome phagocytes, we did not find significant CXCR5 expression in any CD11c^+^ phagocytes. Therefore, identity of CD11c^+^CXCR5^+^ cells in PP as well as the mechanism of transfer of infectious prion from TIM-4^−^ LysoMac to follicular DC remain pending issues.

### Behavior of PP Phagocytes upon Infection

Our knowledge on the alteration induced by pathogens on PP phagocyte populations is scarce. What we know relies mainly on stimulation of PP with pathogen-derived compounds or mimetics. Thus, the cholera toxin induces the migration of CD11c^+^ cells into the FAE. Several TLR ligands induce similar CD11c^+^ cell relocation (112, 134–136). This is in line with the recruitment of DN cDC2 and formation of TMD observed shortly after *Salmonella* infection (43, 117). Thus, the first event that occurs upon pathogen detection is an increase of the sampling activity by recruitment in the FAE of both DN cDC2 and LysoDC. Then, SED-located CD11c^+^ cells are thought to migrate from the SED to the IFR in order to prime naïve T cells. Microsphere-loaded CD11c^+^ cells usually located in the SED are indeed observed in the IFR after cholera toxin or *Salmonella Typhimurium*-induced stimulation (137). Moreover, systemic injection of soluble *Toxoplasma gondii* tachyzoite antigen leads to a loss of CD11c^+^CD11b^+^ cells in the SED combined with the recruitment of CD11c^+^CD11b^+^ cells in the IFR (37). Actually, all activated dome cDC are located in the IFR as exemplified by their specific expression of CD83, CD86, CD205, CCL22, and CCR7 (40). Finally, the number of interfollicular cDC increase in the IFR of R848-fed animals (40, 138). However, this is at least in part due to interfollicular cDC1 number increase and to DAV cDC2 recruitment through a TNF-dependent pathway (40). The respective contribution of DAV and SED cDC2 to the migratory pool of interfollicular cDC is currently unknown, as well as their role in the induction of the mucosal immune response. Nevertheless, DAV and SED cDC recruitment in the IFR upon stimulation may allow in a single region the presentation of antigens sampled both in DAV and in SED. Since uptake of pathogens is facilitated in the FAE as compared to villous epithelium (11, 25, 42, 43), such mechanism of antigen sorting could help the mucosal immune system to discriminate between innocuous and harmful matters. Whether other stimuli than R848 induce similar recruitment of DAV cDC in the IFR is, however, currently unknown. If so, the current model of PP phagocyte activation will have to be modified to include DAV cDC as an integral part of the process.

## Concluding Remarks

Although PP phagocytes are now well characterized, many efforts have to be done in order to understand the role of each phagocyte population in the mucosal immune response initiation during enteric infection. Importantly, to assess carefully these functions, a convenient and well-established panel of markers should be used in the different research laboratories in order to clearly identify each subset and avoid confusion between them. Here, we propose two panels of markers, one for microscopy and one for flow cytometry, which allow distinguishing each PP subset including DAV cDC and DAV MF (Table 1). These panels undoubtedly identify each subset of PP phagocytes and in the future should help clarify their functions in the initiation of the mucosal immune response.

## Author Contributions

HL wrote the manuscript. CDS, CW, JB, and J-PG gave feedback and revised the manuscript.

## Conflict of Interest Statement

The authors declare that the research was conducted in the absence of any commercial or financial relationships that could be construed as a potential conflict of interest.
